# Stevens–Johnson Syndrome and Toxic Epidermal Necrolysis: A Review of Diagnosis and Management

**DOI:** 10.3390/medicina57090895

**Published:** 2021-08-28

**Authors:** Robert Frantz, Simo Huang, Abhirup Are, Kiran Motaparthi

**Affiliations:** 1 College of Medicine, University of Florida, Gainesville, FL 32606, USA; rfrantz@ufl.edu (R.F.); acare2019@hotmail.com (A.A.); 2 Department of Dermatology, Lewis Katz School of Medicine, Temple University, Philadelphia, PA 19140, USA; simo.huang@tuhs.temple.edu; 3 Department of Dermatology, College of Medicine, University of Florida, Gainesville, FL 32606, USA

**Keywords:** Stevens–Johnson Syndrome, Toxic Epidermal Necrolysis, cutaneous adverse drug reactions

## Abstract

Stevens–Johnson Syndrome (SJS) and Toxic Epidermal Necrolysis (TEN) are rare diseases that are characterized by widespread epidermal necrosis and sloughing of skin. They are associated with significant morbidity and mortality, and early diagnosis and treatment is critical in achieving favorable outcomes for patients. In this scoping review, Excerpta Medica dataBASE and PubMed were searched for publications that addressed recent advances in the diagnosis and management of the disease. Multiple proteins (galectin 7 and RIP3) were identified that are promising potential biomarkers for SJS/TEN, although both are still in early phases of research. Regarding treatment, cyclosporine is the most effective therapy for the treatment of SJS, and a combination of intravenous immunoglobulin (IVIg) and corticosteroids is most effective for SJS/TEN overlap and TEN. Due to the rare nature of the disease, there is a lack of prospective, randomized controlled trials and conducting these in the future would provide valuable insights into the management of this disease.

## 1. Introduction

Stevens–Johnson Syndrome (SJS) and Toxic Epidermal Necrolysis (TEN) are dermatologic emergencies characterized by widespread epidermal necrolysis and sloughing. They are considered to have the same pathophysiology and are classified based on body surface area (BSA) involved (Table 1) [1]. These are rare diseases and reported incidence rates vary by location. Frey et al. [2] reported an incidence of 5.76 cases of SJS/TEN per million persons per year in the UK from 1995–2013. Hsu et al. [3] reported 9.2, 1.6, and 1.9 cases per million adults per year in the US from 2009–2012 for SJS, SJS/TEN, and TEN, respectively. Yang et al. [4] reported incidence rates in Korea from 2009–2013 as 3.96–5.03 and 0.94–1.45 per million persons per year for SJS and TEN, respectively. Regarding the pediatric population, Hsu et al. [5] reported an incidence rate of 5.3 and 0.4 cases per million children for SJS and TEN, respectively. Additionally, females are more commonly affected than males at a ratio of approximately 1.5:1 [6,7,8,9,10,11]. The mortality rates are 4.8–9% for SJS, 19.4–29% for SJS/TEN, and 14.8–48% for TEN [3,6].

## 2. Clinical Features

**Figure 1:**
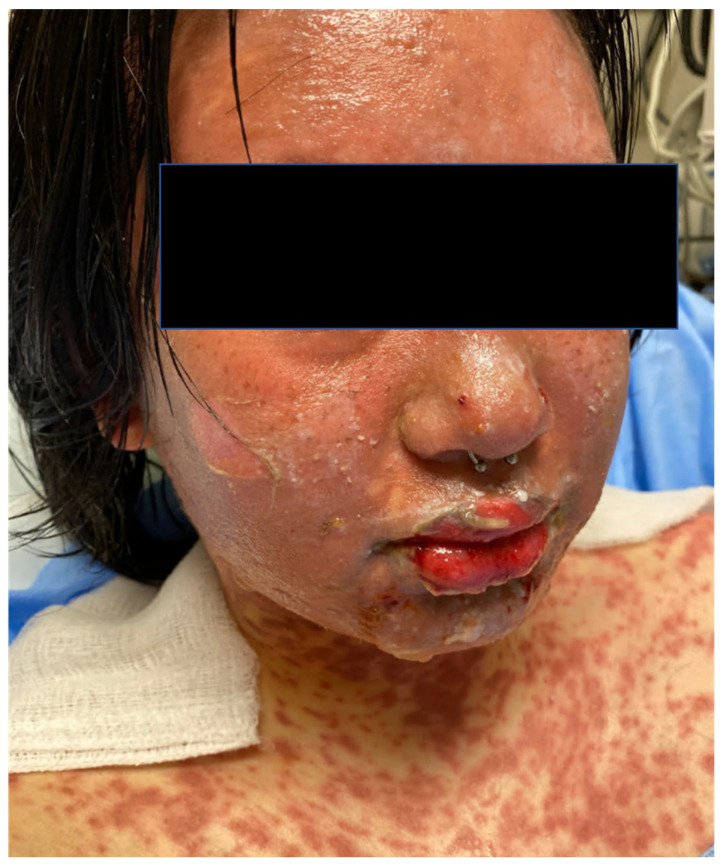
A patient with Toxic Epidermal Necrolysis due to carbamazepine. Dusky macules which reach confluence, along with epithelial detachment on the face and lips.

Clinical features, with the exception of BSA involved, are similar across the disease spectrum. Cutaneous involvement is preceded by a prodromal stage of symptoms, such as fever, malaise, sore throat, and cough in a majority of cases [12,13,14]. Subsequent cutaneous and mucosal involvement is universal and typically appears as erythematous macules or atypical target lesions on the trunk that progress to become confluent areas of erythema with dusky centers, flaccid blisters with a positive Nikolsky sign, and sheets of denuded epidermis [15,16]. The vast majority of patients have mucosal involvement, with two or more mucosal surfaces being involved in up to 80% of cases (Figure 1) [3]. Oral involvement is most common, with mucositis and ulceration occurring in up to 100% of cases [17]. Ocular involvement also occurs frequently, with severity ranging from conjunctival hyperemia to complete epidermal sloughing of the ocular surface. Early consultation with an ophthalmologist is essential to prevent long-term ocular sequelae [13,17,18,19]. Gynecologic involvement also varies in severity but is seen in up to 77% of female patients [17].

## 3. Pathophysiology

Drugs are the most common trigger of SJS/TEN (Table 2), but infection, most commonly *Mycoplasma pneumonia*, has also been implicated [12,15,20,21,22,23,24,25,26,27,28]. In up to 15–30% of cases, no offending agent can be identified [1,29]. While the triggers of these diseases have been well-documented, their pathophysiology has still not been fully elucidated. They are believed to be T-cell-mediated, type IV hypersensitivity reactions. There are a number of hypotheses regarding how drugs generate an immunological response to cause SJS/TEN [30,31,32,33]. The first is the hapten/pro-hapten concept, which states that small-molecule drugs will covalently bind to proteins in serum, forming a complex that is recognized by certain HLA molecules and presented to T-cells to generate an immune response. The next hypothesis, called the pharmacological interaction (p-i) concept, states that chemically inert drugs, which cannot undergo covalent binding with serum proteins, bind HLA molecules directly leading to T cell activation. The final hypothesis is the altered peptide concept, which states that drugs bind inside HLA binding pockets in a way that alters presentation of self-proteins to T cells, such that they are no longer recognized as self, leading to an immune response [30,31,32,33]. Despite uncertainty regarding the exact mechanism, the end result is activation of T-cells in response to a drug or infection and downstream epidermal necrosis.

Early hypotheses postulated that keratinocyte death was mediated by soluble Fas ligand (sFasL) interactions with the Fas receptor on the surface of keratinocytes [34]. Subsequent studies identified granulysin as a more important mediator of apoptosis. Chung et al. [35] analyzed the blister fluid of SJS/TEN patients and found that granulysin levels were 2–4 times higher than perforin, granzyme B, and sFasL. Additionally, reducing granulysin levels reduced cytotoxicity and injection of granulysin into the skin of mice induced an SJS/TEN-like reaction [35]. Further studies confirmed the role of granulysin as a major mediator of the disease and showed that the levels of granulysin in blister fluid correlated with the severity of the disease [36,37,38]. While granulysin seems to be the main driver of epidermal necrosis, it does not act alone. Su et al. [39] examined the serum levels of 28 different cytokines and chemokines and found a number that were upregulated in patients with SJS/TEN, of which granulysin and IL-15 correlated with the severity of the disease. Additionally, the role of necroptosis, or programmed necrosis, has been examined and was found to contribute to keratinocyte death, which could have important diagnostic implications [30,40,41,42].

## 4. Differential Diagnosis

**Figure 2:**
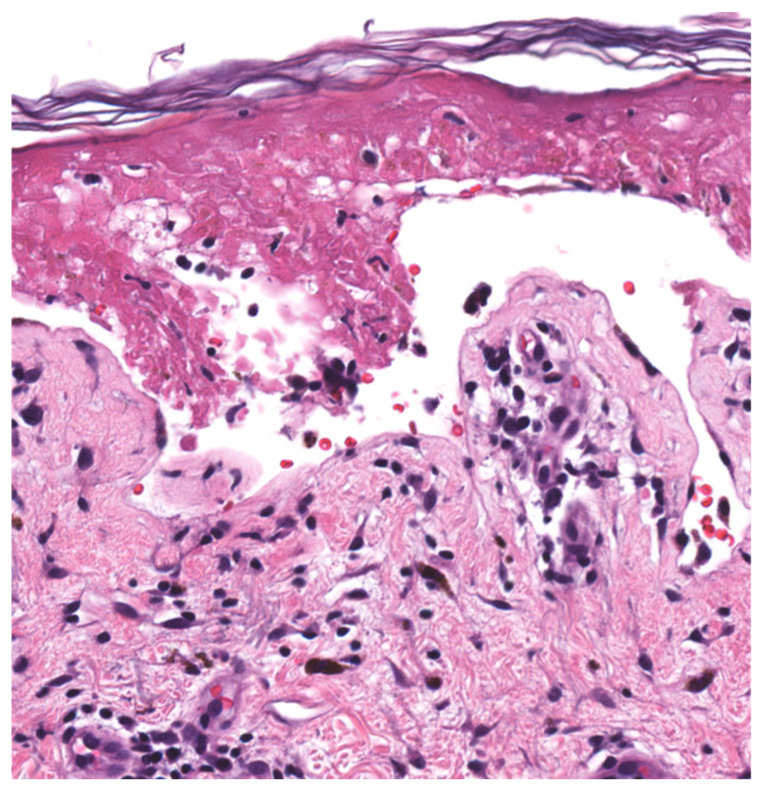
Histopathology of Stevens–Johnson Syndrome. Full thickness epidermal necrosis, an orthokeratotic stratum corneum, and sparse dermal inflammation are typical and supportive but nonspecific features.

Prior to diagnosis of SJS/TEN, a broad differential diagnosis may be considered (Table 3). This includes other desquamating and vesiculobullous dermatoses, such as pemphigus vulgaris, linear IgA bullous dermatosis, staphylococcal scalded skin syndrome (SSSS), and erythema multiforme major (EMM). Importantly, EMM and SJS/TEN were historically classified as existing on the same disease spectrum, as the clinical and histopathologic presentation (Figure 2) of these diseases can be similar, but were later determined to be distinct diseases [6,13,14,15,16]. Thus, the diagnosis must be made on clinical parameters. Key features of these disorders are outlined in Table 4 [1,6,13,14,15,16,43,44,45].

## 5. Management

The management of SJS/TEN is multifaceted and begins with identification and cessation of the causative agent [46]. A thorough history is important to identify the causative agent [47], as symptoms typically present within 8 weeks of beginning therapy, with most cases appearing between 4 days and 4 weeks of starting a drug [12]. If history is not sufficient to ascertain the causative drug, a number of causality assessment tools (CATs) can be useful. The Algorithm for Drug Causality for Epidermal Necrolysis (ALDEN) [29] and the Liverpool Adverse Drug Reaction CAT [48] are algorithms that have proven to be effective identifiers of causative drugs. The lymphocyte transformation test (LTT) is an in vitro test that can detect sensitization of T cells to antigens and can be helpful in identification of causative drugs in SJS/TEN, although at this time it is largely used for research purposes [45].

Prognostication is also an important step in the management of SJS/TEN, as it can guide management and placement in an intensive care or burn unit [49]. The severity-of-illness score for Toxic Epidermal Necrolysis (SCORTEN) scale is the most widely used tool for determining prognosis in patients with SJS/TEN. This has been verified as an effective tool in a number of studies [50,51]. Other studies, however, have shown that SCORTEN may overestimate actual mortality rates [52,53]. However, this discordance may potentially be attributed to improvements in supportive care since the development of SCORTEN in 1979 [51]. Noe et al. [54] developed an alternative prognostic algorithm called ABCD-10. This scoring system uses prior dialysis as a proxy for severe renal dysfunction, distinguishing it from SCORTEN. Both scoring systems seem to be reliable predictors of mortality, but one study [55] showed that SCORTEN was more accurate. The SCORTEN and ABCD-10 scoring systems and predicted mortality are outlined in Table 5 and Table 6. One important note for both scoring systems is how to determine the BSA involved, as an accurate estimate is critical for classification and prognostication. Creamer et al. [56] described that BSA involved includes both epidermis that is detachable (positive Nikolsky sign) and epidermis that is already detached. Areas of erythema without evidence of detachment or impending detachment are not included.

Removal of the offending agent and supportive care are the mainstays in treatment of SJS/TEN [57]. Adjunctive therapies, such as corticosteroids and intravenous immunoglobulin (IVIg), are often utilized, although there is still no consensus on the most effective adjunctive therapy. The goal of this article is to review the most recent updates in both diagnosis and management of SJS/TEN in order to educate dermatologists and other physicians who are managing the acute care of patients with SJS/TEN.

## 6. Materials and Methods

A database search of PubMed and Embase was performed, initially focusing on review articles in the past 5 years, from March 2017 through March 2021, with keywords “Stevens–Johnson Syndrome”, “Toxic Epidermal Necrolysis”, “therapy”, “diagnosis”, “management”, and synonyms of all these key words. The reference section of each of the review articles was also reviewed to find other articles that contained pertinent information.

## 7. Clinical Updates

### 7.1. Updates on Diagnosis

#### 7.1.1. Potential Biomarkers

Rapid diagnosis of SJS/TEN is critical in order to discontinue the offending agent, begin supportive and adjunctive therapies, and improve outcomes. However, the clinical presentation can be similar to a number of other blistering disorders, and diagnosis is not always straightforward. Given that the diagnosis of SJS/TEN is time sensitive, frozen sections can be utilized for more rapid decision making. SJS/TEN can be distinguished from SSSS by the level of epidermal detachment, which is subcorneal in SSSS and at the dermo-epidermal junction in SJS/TEN. Widespread keratinocytic necrosis is characteristic of SJS/TEN on histopathology [43,58]. The distinction between SJS/TEN and EMM is difficult to make because their histopathology can be identical [13,16]. In the early stage of both diseases, a vacuolar or lichenoid interface with scattered necrotic keratinocytes can be seen. As both diseases progress, a subepidermal split with increased epidermal necrosis is observed. In these cases, a heavier lymphocytic infiltrate favors EM while increased eosinophils and confluent epidermal necrosis favors SJS/TEN. However, these are not reliable distinguishing features and clinicopathologic correlation is required.

There are a number of studies that have investigated potential diagnostic markers (Table 7) of the disease, with early studies focusing on the role of granulysin. Abe et al. [37] analyzed the serum of 5 patients with SJS/TEN and found elevated levels of granulysin in 4 out of 5 patients, even before cutaneous detachment and mucosal involvement. Sera from thirty-one control patients were also analyzed and showed no elevations in serum granulysin levels. Chen et al. [36] found that granulysin levels in blister fluid were markedly elevated and correlated with disease severity in SJS/TEN. However, these findings were consistent across all cytotoxic T-lymphocyte (CTL)-mediated bullous blistering disorders, such as EMM and bullous fixed drug eruption (BFDE). Elevated serum granulysin levels were also observed in patients with drug reaction with eosinophilia and systemic symptoms (DRESS) [38]. Therefore, while granulysin is elevated in both serum and blister fluid, it is not a specific finding for SJS/TEN and has limited utility in early diagnosis.

CCL-27 is another nonspecific cytokine that is likely involved in the pathogenesis of SJS/TEN and aids in the trafficking of T cells to the skin at sites of inflammation [30]. Tapia et al. [59] reported that CCL-27 levels were elevated in skin from patients with SJS/TEN during the acute phase. Wang et al. [60] then analyzed the levels of CCL-27 in sera from 27 patients with SJS/TEN and found elevations during the acute phase compared with 39 healthy controls. This implicates CCL-27 in the pathogenesis of SJS/TEN, but elevated CCL27 levels were also identified in non-bullous drug-induced exanthems. Therefore, the use of CCL-27 in diagnosis is limited in the same manner as granulysin, due to the lack of specificity.

There are a number of other potential biomarkers under investigation that may demonstrate specificity for SJS/TEN. In one study [61], peripheral blood mononuclear cells (PBMCs) from patients who had recovered from SJS/TEN were cultured and re-exposed to the causative drug. The supernatant of the culture fluid was analyzed using proteomics to identify potential biomarkers. This protocol was also used to evaluate the molecules secreted by PBMCs in non-severe cutaneous adverse drug reactions (cADRs). When comparing the two groups, Hama et al. [61] discovered one protein, galectin-7, exhibited higher levels in sera of SJS/TEN patients than in sera of non-severe cADRs (*p* = 0.005). Serum galectin-7 also correlated with disease severity with significantly higher levels during the acute phase and decreased levels in the late phase of the disease (>7 days). Galectin-7 could be a potential mediator of SJS/TEN and a helpful biomarker for diagnosis. 

The role of necroptosis, or programmed necrosis, in SJS/TEN has been the focus of multiple studies. Necroptosis differs from apoptosis in that cell death is the result of external triggers that alter membrane permeability and result in cell lysis without the involvement of caspases. Recent studies have identified receptor-interacting kinase-3 (RIP3) as an important mediator [41,62]. Hasegawa et al. [41] confirmed that necroptotic keratinocytes release RIP3 into the sera of patients, and its levels correlated with the degree of necroptosis and severity of disease. Notably, the investigators also measured RIP3 levels in the sera of patients with EMM and found significantly higher levels in patients with SJS/TEN than EMM (*p* < 0.001). The use of serum RIP3 as a biomarker for the diagnosis of SJS/TEN could help distinguish between SJS/TEN and EMM.

#### 7.1.2. Diagnostic Subclassification in Pediatric Patients

The diagnostic classification for pediatric patients has been recently updated. Canavan et al. [63] performed a systematic review of 202 patients with an SJS/TEN-like reaction to *Mycoplasma pneumoniae* infection. They noted that these patients had impressive mucosal involvement, but the cutaneous involvement was less significant and the prognosis more favorable compared with SJS/TEN. Canavan et al. named this dermatosis *Mycoplasma pneumoniae*-induced rash and mucositis (MIRM) and classified it as distinct from SJS/TEN and EM. Subsequently, multiple other studies implicated other infections as causes of MIRM-like reactions including adenovirus [64], influenza B [65], and *Chlamydia pneumoniae* [66].

In light of these findings, Ramien et al. [67] proposed a new classification for blistering disorders in pediatric patients. In this new system, SJS, SJS/TEN, and TEN are condensed into a single disorder called drug-induced epidermal necrolysis (DEN). The infection-related cases with severe mucosal involvement and relatively sparse cutaneous involvement were considered a distinct clinical identity and termed reactive infectious mucocutaneous eruption (RIME). Erythema multiforme (EM) was classified as a distinct disease from DEN and RIME [68]. This new classification is worthwhile because the treatment of DEN and RIME differ. In DEN, identification and cessation of the causative drug with supportive care and possible immunosuppressive therapy are the pillars of treatment. RIME requires identification and treatment of the underlying infection, supportive care, and potential antimicrobial and immunosuppressive therapies. In fact, the use of antibiotics to treat community acquired pneumonia in patients with RIME has been emphasized [69,70]. One study examined the role of etanercept treatment in RIME and showed that this therapy led to improvement in physical findings within 2 days of drug administration [71]. However, this study is limited by its small sample size (*n* = 6) and treatment with antibiotics in 5/6 patients prior to administration of etanercept, which could also have contributed to the observed improvement. Further studies are required to clarify proper treatment strategies for both DEN and RIME.

### 7.2. Updates on Management

#### 7.2.1. Non-Pharmacologic Treatment

Supportive care is the mainstay of treatment for patients with SJS/TEN and includes cessation of the causative drug, fluid and electrolyte management, infection control, and wound care. Of these components, identification and cessation of the causative drug is most important [46,49,72], but optimization of each measure is necessary to achieve the best outcomes.

Fluid, electrolyte, and nutrition management is important in SJS/TEN patients and mirrors the requirements of burn patients due to insensible losses through wounds, although fluid requirements are about 30% less in SJS/TEN patients than in burn patients with similar degrees of cutaneous involvement [73,74]. The environment should be kept warm (30–32 °C) [49] due to loss of thermoregulatory function of skin, and fluid replacement should be driven by urine output, with a goal of 0.5–1 mL/kg/h [73]. Enteral feeding should be initiated as early as possible and through nasogastric tube feeds if necessary [49].

Prophylactic antibiotics do not improve outcomes [75], but proper wound care and sterile handling are important in preventing infection. The role of surgical debridement has been controversial, and the decision to pursue this treatment option largely depends on where the care is being delivered. McCullough et al. [57] described a series of 40 SJS/TEN patients who were treated with their treatment algorithm, which included aggressive supportive care, surgical wound debridement with subsequent coverage with antimicrobial dressings, steroid cessation (if the patient was receiving steroids upon transfer), and IVIg. The authors of this study reported a 10% mortality rate, which was lower than the 16.7% mortality rate predicted by SCORTEN. While this result reflects an effective combination of treatments, it is difficult to identify surgical debridement as the cause of that efficacy. Dorafshar et al. [76] analyzed the efficacy of “anti-shear” therapy, in which blister fluid is aspirated and denuded epidermis is left in place to act as a biological skin graft. The authors described 48 patients at their care center who received this treatment and presented a mortality reduction of 11 percent compared to the expected mortality predicted by SCORTEN. Anti-shear therapy is an alternative to surgical debridement and could reduce hospital costs as well as pain. However, there is a lack of high-quality evidence to guide decision-making regarding surgical debridement [77], and further studies are needed to fully understand the role of this therapy.

#### 7.2.2. Pharmacologic Treatment

Due to the rarity of the disease, few prospective studies have analyzed the efficacy of specific adjunctive therapies for SJS/TEN. As a result, there is no established standard of care pertaining to pharmacologic treatment. Due to the immunologic nature of the disease, it is believed that immunosuppressive therapies will aid in treatment, and many case reports have reported positive results with varying treatment regimens involving different combinations of corticosteroids, IVIg, cyclosporine, and TNF-alpha inhibitors [21,78,79,80,81]. However, it is difficult to determine if disease remission was due to specific treatment or simply the natural history of the disease. Several systematic reviews and meta-analyses have attempted to overcome these methodologic limitations and clarify the role of pharmacologic therapies in the treatment of SJS/TEN.

The role of corticosteroids as monotherapy is still debated [82]. Recently, Zimmermann et al. [83] performed a meta-analysis of 11 studies to compare the use of corticosteroids versus supportive therapy and found a positive, although statistically insignificant (OR, 0.54; 95% CI, 0.29–1.01), treatment effect. Other studies have shown no improvement in mortality with the use of corticosteroids alone [10,30]. The role of IVIg has also been controversial, and there appears to be no mortality benefit associated with monotherapy [56,58,84,85].

Despite uncertainty in the results of these therapies, there are a number of other treatment options that show promise. Cyclosporine has shown positive results in a number of studies to this point [83,86,87,88,89]. Ng et al. [88] performed a meta-analysis of 10 studies and reported on the standardized mortality ratio (SMR) of cyclosporine compared with supportive care. The SMR takes into account baseline severity of the disease, allowing for a more accurate depiction of mortality improvement as compared to mortality ratios (MR). In this study, the authors reported an SMR of 0.320 (95% CI, 0.119–0.522, *p* = 0.002), indicating a survival benefit in patients treated with cyclosporine. Chen et al. [89] performed a meta-analysis of 7 studies and reported similarly positive results with an SMR of 0.42 (95% CI, 0.19–0.95) when cyclosporine was administered.

In another study, Tsai et al. [90] analyzed treatment outcomes of a number of therapies and performed a meta-analysis of 67 studies involving 2079 patients. The authors only examined the mortality outcomes of patients with SJS/TEN overlap and TEN, choosing to exclude SJS outcomes because mortality is typically lower. The only therapy that showed statistically significant improvements in outcomes was the combination of IVIg and corticosteroids, with an SMR of 0.53 (95% CI, 0.31–0.93). Historically, IVIg has been used as a monotherapy [91], but it only appears to be effective when combined with corticosteroids. These authors also reported promising results for cyclosporine (with or without IVIg), IVIg and plasmapheresis, and etanercept, although they emphasized the need for further studies.

Han et al. [92] performed a prospective observational study of 28 patients with SJS/TEN overlap or TEN, 13 of whom received plasmapheresis and 15 of whom did not. Of the 13 that received plasmapheresis, 7 were also treated with concomitant corticosteroids or IVIg. Using a severity of illness score that evaluated mucosal lesions, cutaneous lesions, and overall general condition (scores 0–39), it was shown that patients who received plasmapheresis had a lower severity of illness scores later in the disease course (days 7, 10, and 20).

TNF-alpha inhibitors are also of interest due to their immunosuppressive effects. Zhang et al. [93] reviewed 21 case reports, 4 case series, and 2 randomized controlled trials (RCTs) that analyzed the use of TNF-alpha inhibitors and reported positive outcomes in 86.8% of patients. One of these RCTs [94] included 91 patients and showed improvement in mortality. The observed mortality rate of 8.3% was lower than that predicted by SCORTEN (17.7%) and lower than the mortality associated with corticosteroid treatment (16.3%), although these results were not statistically significant.

Given the lack of consensus on the most effective pharmacological treatment for SJS/TEN, practical issues such as cost must also be taken into account when determining treatment course. Table 8 and Table 9 outline practical considerations for these drugs, including dosing regimens and costs.

## 8. Discussion

SJS/TEN is a dermatologic emergency that causes significant morbidity and mortality. Early in the disease course, there is a broad differential diagnosis that needs to be considered, and prompt diagnosis is critical in achieving optimal outcomes. For most of the potential differential diagnoses, clinical morphology and histopathology can readily distinguish them from SJS/TEN. However, EMM is a disease that has identical histopathological features to SJS/TEN, and confident distinction between these two disorders requires experience and more careful correlation. SJS/TEN has significantly higher mortality and morbidity rates than those of EMM, often necessitating surgical or medical therapy beyond supportive care alone. A number of serological tests show promise in expanding the ability to diagnose SJS/TEN. Granulysin and CCL-27 serum markers are elevated in patients with SJS/TEN and can be helpful markers to monitor disease severity. However, these markers are not specific for SJS/TEN and are elevated in other disorders, limiting their specificity. Both galectin-7 and RIP3 play a pathogenic role and are elevated to a greater degree in the sera of patients with SJS/TEN compared to other cADRs. Further research is required before these markers can be reliably used for diagnosis.

Once diagnosed, the management of SJS/TEN focuses primarily on supportive care and wound management with the addition of adjunctive medications. The role of surgical debridement has been debated, and evidence has shown that both surgical debridement and anti-shear therapy improve patient outcomes. Wound management and infection prevention improve outcomes, as both therapies are effective. Corticosteroids, IVIg, cyclosporine, TNF-alpha inhibitors, and plasmapheresis are therapeutic options. Variable results have been described, and there is still no consensus on the treatment of choice. Few RCTs have been conducted, and most of the research published has been in the form of case reports, case studies, and systematic reviews and meta-analyses. The most efficacious treatments appear to be cyclosporine and a combination of corticosteroids with IVIg. Both have shown statistically significant improvements to patient mortality. It is also important to consider cost effectiveness when selecting therapies. Of the drugs described in the treatment of SJS/TEN, the most expensive is infliximab, followed by IVIg and etanercept. The least expensive options are cyclosporine and corticosteroids. Ultimately, further prospective studies are required to solidify treatment guidelines.

## Figures and Tables

**Table 1:** Diagnosis of Stevens-Johnson Syndrome (SJS) and Toxic Epidermal Necrolysis (TEN) based on body surface area (BSA) (%) involvement.

Diagnosis Based on BSA (%)	
SJS	<10%
SJS/TEN Overlap	10–30%
TEN	>30%

**Table 2:** Common drugs implicated in the pathogenesis of SJS/TEN.

Common Drug Triggers of SJS/TEN	
Anti-epileptics	Antibiotics
○ Lamotrigine	○ TMP-SMX
○ Phenytoin	○ Aminopenicillins
○ Carbamazepine	○ Tetracyclines
○ Valproic Acid	○ Cephalosporins
○ Phenobarbital	Immune Checkpoint Inhibitors
NSAIDs	○ Nivolumab
Allopurinol	○ Pembrolizumab
Nevirapine	

**Table 3:** Differential diagnosis of suspected SJS/TEN.

Differential Diagnosis of SJS/TEN	
Erythema multiforme major	Pemphigus vulgaris
Staphylococcal scalded skin syndrome	Bullous pemphigoid
Generalized fixed drug eruption (BFDE)	Linear IgA bullous dermatosis
Acute generalized exanthematous pustulosis	Paraneoplastic pemphigus
Phototoxic eruptions	Acute or subacute cutaneous lupus with epidermal necrosis (Rowell syndrome)

**Table 4:** Distinguishing characteristics of SJS/TEN and EM.

SJS/TEN vs. EM		
	SJS/TEN	EM
Characteristic Lesions	Atypical target lesions: macules with central clearing and 2 poorly demarcated components	Typical target lesions: papules with a dark center and 3 well-demarcated, concentric components
	Large sheets of painful desquamation in later lesions	
Distribution	Typically begins on the face and trunk with centrifugal spread	Face and acral skin, rare involvement of trunk
Triggers	Drugs (see Table 2)	Infection (most commonly HSV and *M. pneumonia*)
Mucosal Involvement	Very common—most cases have involvement of ≥2 mucosal surfaces	Rare—typically only one mucosal surface involved if present
Recurrence	Rarely seen with removal and avoidance of causative drug	Frequently seen
Histopathology (Figure 2)	Early Stage Basal layer liquefaction with vacuolar interface changes, scattered necrotic keratinocytes, and interface lymphocytes	
	Late Stage * Subepidermal split with full-thickness epidermal necrosis	

* Biopsy in the late stages of SJS/TEN may show comparatively little inflammation compared to EM.

**Table 5:** SCORTEN and ABCD-10 Scoring Systems.

SCORTEN		ABCD-10	
Parameter	Weight	Parameter	Weight
Age ≥ 40 years	1	Age ≥ 50 years	1
Malignancy—Yes	1	Serum Bicarbonate < 20 mmol/L	1
BSA detached > 10%	1	Active Cancer—Yes	2
Serum bicarbonate < 20 mmol/L	1	Dialysis prior to admission—Yes	3
Serum urea nitrogen > 28 mg/dL	1	BSA Involvement > 10%	1
Serum glucose > 252 mg/dL	1		
Tachycardia ≥ 120 bpm	1		
Maximum score possible	7		8

**Table 6:** Estimated mortality in patients with SJS/TEN.

Estimated Mortality in Patients with SJS/TEN			
SCORTEN Score	Estimated Mortality (%)	ABCD-10 Score	Estimated Mortality (%)
0–1	3.2	0	2.3
2	12.1	1	5.4
3	35.3	2	12.3
4	58.3	3	25.5
>5	>90	4	45.7
		5	67.4
		>6	83.6

**Table 7:** Potential Biomarkers for the Diagnosis of SJS/TEN

Common Drug Triggers of SJS/TEN	
Non-Specific for SJS/TEN	Specific for SJS/TEN
○ Granulysin ○ CCL-27	○ Galectin-7 ○ RIP3

**Table 8:** Typical dosing regimens to treat SJS/TEN for selected drugs

Dosing Regimen for SJS/TEN of Selected Drugs	
IVIg	3 g/kg, divided over 3 days [90]
TNF-alpha inhibitors	- Infliximab: 5 mg/kg as a single dose [92] - Etanercept: Single 50 mg dose [92]
Cyclosporine	2.5–5 mg/kg/day for 7–10 days, followed by gradual taper [87,88]
Corticosteroids	Prednisone 0.5–1 mg/kg/day or pulse methylprednisolone 1 mg/kg/d for 3 days [81]

**Table 9:** Relative cost of selected drugs at a single academic center

Relative Cost of Selected Drugs **	
IVIG	$1932 for a treatment course *
Etanercept	$1386 for a single 50 mg subcutaneous dose
Infliximab	$4900 *
Cyclosporine	~$336 for a 3-week course/taper at $16 per day
Prednisone	<$20 for 2–3-week taper at $1 per day

* Assuming a 70 kg individual. ** Cost and access may vary by medical center.
